# Association between PM_2.5_ constituents and non-suicidal self-injury among Chinese adolescents: modifying role of biological rhythm

**DOI:** 10.3389/fpubh.2026.1887542

**Published:** 2026-08-20

**Authors:** Xiaodi Ren, Longqing Ouyang, Yang Xie, Shuqin Li, Runyu Wei, Yi Zhang, Huiqiong Xu, Hui Gao, Fangbiao Tao, Yuhui Wan, Ying Tang

**Affiliations:** 1 Department of Maternal and Child Nursing, School of Nursing, Anhui Medical University, Hefei, Anhui, China; 2 Key Laboratory of Population Health Across Life Cycle (Anhui Medical University), Ministry of Education, Hefei, Anhui, China; 3 Ma'anshan Maternal and Child Health Center, Ma'anshan, Anhui, China; 4 School of Public Health, Anhui Medical University, Hefei, Anhui, China; 5 Department of Endocrinology, The First Affiliated Hospital of Anhui Medical University, Hefei, China; 6 School of Public Health, Anhui Institute of Medicine, Hefei, Anhui, China; 7 Department of Pediatrics, The First Affiliated Hospital of Anhui Medical University, Hefei, Anhui, China

**Keywords:** adolescents, biological rhythms, constituents, non-suicidal self-injury, PM2.5

## Abstract

**Background:**

Non-suicidal self-injury (NSSI) has emerged as a growing public health concern among Chinese adolescents. Although exposure to fine particulate matter (PM_2.5_) has been associated with adverse mental health outcomes, the specific chemical constituents driving this association and the potential modifying role of biological rhythms remain poorly understood.

**Methods:**

A multicenter, population-based survey was conducted across 15 schools located in four provinces of China. A total of 18,398 adolescents in grades 7 through 12 completed the eligible questionnaires. NSSI behaviors were assessed using the Adolescent Non-suicidal Self-injury Assessment Questionnaire (ANSAQ). The Self-rating Questionnaire of Biological Rhythm Disorders for Adolescents (SBRDA) was used to conduct a comprehensive assessment of participants’ multidimensional biological rhythms. Binary logistic regression and weighted quantile sum (WQS) regression were employed to examine the associations between PM_2.5_ constituents and NSSI, with stratified analyses performed to explore the moderating effects of factors such as biological rhythms.

**Results:**

After adjusting for covariates, PM_2.5_ and its constituents showed significant positive associations with NSSI risk, with black carbon exhibiting the strongest association (*OR* = 1.76, 95% *CI*: 1.60–1.94). Mixed exposure analyses consistently identified black carbon as the dominant constituent (WQS weight = 0.810; Qgcomp effect size = 0.671). Stratified analyses revealed heightened vulnerability among adolescents with disrupted biological rhythms, good family economic situations, junior high school enrollment, and heavy learning burdens. Furthermore, disruptions in activity rhythm and eating rhythm can significantly amplify the risk impact of pollutants on NSSI behaviors.

**Discussion:**

Exposure to PM_2.5_ (particularly BC) increases adolescent NSSI risk, with factors like biological rhythm disruption potentially amplifying this effect. Integrating strategies to manage BC emissions with initiatives that encourage adolescents to maintain healthy routines, such as timely meal consumption, could synergistically lower mental health risks.

## 1 Introduction

Non-suicidal self-injury (NSSI) refers to intentional and direct harm inflicted on one’s body without the intention of suicide, including actions like cutting, burning, biting, and scratching the skin (1, 2). Although NSSI does not directly cause death, it has been identified as a significant predictor of suicide (3). Adolescence is an important phase for the emergence and progression of NSSI. Epidemiological studies indicate that 17–20% of adolescents worldwide report having engaged in NSSI (4). A comprehensive review of 256 studies found that the lifetime prevalence of NSSI among Chinese adolescents is estimated to reach 24.7% (*N* = 1,088,433) (5). This prevalence rate surpasses the global average by a significant margin, indicating that NSSI is becoming an increasingly important public health concern among Chinese adolescents.

For an extended period, etiological studies on adolescent NSSI have largely focused on personal attributes, childhood experiences and social influences (6–8). Recently, within the environmental exposomics framework, research increasingly links fine particulate matter (PM_2.5_) pollution to mental health issues in adolescents, highlighting it as a major global environmental health concern (9, 10). PM_2.5_ generally consists of a mixture of carbon-based fractions, earth elements, soluble ions, metals, and other constituents (11). PM_2.5_ and its constituents may increase NSSI risk by causing neuroinflammation and oxidative stress, and by penetrating the blood–brain barrier, damaging brain areas crucial for emotion and behavior regulation (12–14). Initial cross-sectional studies have already established an association between the two variables (15). Nonetheless, current studies on the link between PM_2.5_ and adolescent NSSI exhibits significant limitations. The majority of research heavily depends on the overall mass concentration of PM_2.5_ while overlooking the distinct toxic effects potentially associated with different chemical constituents such as black carbon (BC), ammonium (NH_4_^+^), nitrate (NO_3_^−^), organic matter (OM), sulfate (SO_4_^2−^). In fact, certain constituents within PM_2.5_, such as BC from open burning and controlled combustion. Due to its porous structure and extensive surface area, it easily absorbs and transports various toxic substances produced during combustion, like polycyclic aromatic hydrocarbons and heavy metals, into the human body, potentially exhibiting more pronounced neurotoxic potential (16, 17). Earlier research has indicated that contact with PM_2.5_ constituents is linked to various negative health effects, but its specific association with adolescent NSSI behavior remains unclear. Furthermore, most studies on environmental exposure among adolescents focus primarily on exposure at school, neglecting the influence of the home environment.

It is worth noting that the impact of environmental pollutants on adolescents’ mental health may be modulated by individual biological rhythms. During adolescence, the increased secretion of gonadal hormones can induce phase delays in the rhythm system and the sleep–wake cycle, rendering the biological rhythm system more susceptible to disruption (18). Biological rhythms encompass not only the sleep–wake cycle but also multidimensional behavioral patterns such as eating habits, physical activity, and the use of digital media. From mechanistic perspective, the biological rhythm may influence the body’s susceptibility to environmental neurotoxins by regulating neuroinflammation and the function of the hypothalamic–pituitary–adrenal axis (19, 20). Previous studies have shown that biorythmic factors such as sleep, physical activity and problematic mobile phone use are closely linked to mental health issues (21, 22). However, there is currently a lack of comprehensive assessment of the modulatory effects of biological rhythms.

To address existing research gaps, this study is the first to utilize large-scale, multicenter, school-home collaborative data from Chinese adolescents to comprehensively investigate the association between PM_2.5_ and its constituents with adolescent NSSI. We propose the following hypotheses: (1) Extended exposure to the overall PM_2.5_ mass concentration is linked to an increased risk of NSSI in adolescents; (2) Among PM_2.5_ constituents, BC, produced by incomplete combustion and highly neurotoxic, shows the strongest correlation with this risk; (3) Regular biological rhythms may mitigate the neurotoxic effects caused by exposure to PM_2.5_ constituents by maintaining neuroendocrine homeostasis and regulating stress. Findings from this study may provide novel scientific evidence for preventing extreme psychological behaviors among adolescents through environmental governance and behavioral adjustments.

## 2 Methods

### 2.1 Study population

Researchers collected data for this study from a Chinese national cohort of adolescent health between November and December 2023. The study encompassed junior high and senior high schools in four cities: Shenzhen in Guangdong Province, Zhengzhou in Henan Province, Zhaotong in Yunnan Province and Taiyuan in Shanxi Province. The study population was selected using cluster sampling, employing the standardized methodology detailed in the research protocol (23, 24). Ultimately, 19,100 students from 15 schools were asked to anonymously complete the questionnaire. After accounting for absences and refusals, 18,440 questionnaires were returned, yielding a 96.55% recovery rate. Following the exclusion of invalid responses, 18,398 valid questionnaires were analyzed, achieving a 96.32% effective response rate (Supplementary Figure S1). The sample included 9,385 males and 9,013 females, with an average age of 15.49 years. The Ethics Review Committee of Anhui Medical University approved this study (Approval No.: 20200965), and informed consent was obtained from students and their parents or guardians.

### 2.2 Non-suicidal self-injury

Every participant was surveyed with the Adolescent Non-suicidal Self-injury Assessment Questionnaire (ANSAQ) (25), which asked if they had intentionally harmed themselves in the past 12 months without suicidal intent. Self-harm methods included pinching, scratching, head-banging, punching, hitting, biting, hair-pulling, pricking, cutting, burning, rubbing skin, and etching on skin. For participants with self-injury, the frequency is recorded, with the total number episodes counted. This study defines “none” as no NSSI, and “one or more” as having NSSI.

### 2.3 Environmental exposure

Data on PM_2.5_ and its five monitored constituents (BC, NH_4_^+^, NO_3_^−^, OM, SO_4_^2−^), along with O_3_ levels, were sourced from the Tracking Air Pollution in China^1^ (26–28). This dataset combines PM_2.5_ monitoring, satellite data, air quality simulations, meteorological reanalysis, land use, elevation, and population data. PM_2.5_ constituents were estimated using an extreme gradient enhancement algorithm at a 10 km × 10 km resolution. During the survey, we gathered participants’ home and school addresses. The process to match these with environmental data involved: converting addresses to geographic coordinates using Baidu Maps, transforming these to the WGS84 system, using ArcGIS and R for spatial analysis to extract pollutant data from the TAP dataset. Finally, the annual average concentrations of PM₂.₅ and its constituents at home and school during the year preceding the survey were calculated to serve as the individual exposure levels at home (*C* home) and at school (*C* school).

To better capture adolescents’ pollutant exposure at home and school, this study used a time-weighted approach to calculate annual exposure concentrations. Adapting methods from the London SCAMP study (29). The following assumptions were made regarding adolescents’ daily time allocation: based on our exposure period, we can calculate that the time spent by teenagers on schoolwork is 198 days, and the time spent at home is 167 days. On school days, they were at school for 8 h and home for 16 h; on non-school days, they stayed home all day. Using this time allocation, environmental weightings were calculated. In this study, PM_2.5_ and its constituents were analyzed as continuous variables and as binary variables (low exposure group/high exposure group) based on the *P*_75_ cutoff.

*C*_weighted_ = (Home_hours / Total_hours) × *C*_home_ + (School_hours / Total_hours) × *C*_school_

Normalized Difference Vegetation Index (NDVI) data is sourced from the National Aeronautics and Space Administration^2^. Nighttime light exposure data is sourced from the Earth Observation Group under the National Centers for Environmental Information of the National Oceanic and Atmospheric Administration^3^. Participants’ NDVI data is weighted using their home and school addresses from the baseline questionnaire. Nighttime light exposure is calculated using their home address.

### 2.4 Biological rhythm

The Self-rating Questionnaire of Biological Rhythm Disorders for Adolescents (SBRDA) was used to assess participants’ actual biological rhythm status over the past 30 days using 29 items across sleep, eating, activity, and digital media usage dimensions (30). It comprises 29 items across four dimensions: sleep rhythm, eating rhythm, activity rhythm, and digital media usage. Each item is rated on a 5-point Likert scale from 1 (“Not at all true”) to 5 (“Completely true”). The sum of items within each dimension constitutes the dimension scores, and the total BRDA score, spanning from 29 to 145, is the aggregate of these dimension scores, where higher scores reflect greater disruption of biological rhythms. The SBRDA’s Cronbach’s alpha was 0.898. Data analysis categorized scores into normal or disordered groups based on the *P*_75_ cutoff.

### 2.5 Covariates

Based on prior literature (31, 32), to assess the independent effects of PM_2.5_ and its constituents on NSSI, the selected key variables include: (a) Sociodemographic factors: grade level (junior high school or high school), gender (boy or girl), residence (rural or town), only child status (yes or no), father’s educational level (primary school or below, junior high school, high school or technical secondary school, college degree or above), mother’s educational level (primary school or below, junior high school, high school or technical secondary school, college degree or above), family’s economic situation (poor, medium, good), close peers (0, 1–2, 3–5, 6 or more), learning burden (medium and below, heavy), and life behind (yes or no); (b) Behavioral factor: biological rhythms (normal, disorder). (c) Environmental factors, including O₃, NDVI, and nighttime light exposure levels.

### 2.6 Statistical analysis

(1) Descriptive statistics and correlation analysis involved using frequencies or percentages for categorical variables and means with standard deviations for continuous variables like pollutant concentration. Spearman’s correlation was used to assess variable relationships. (2) Restricted cubic splines (RCS) with three nodes at the 10th, 50th, and 90th percentiles were used to explore nonlinear relationships between PM_2.5_, its constituents, and NSSI. Likelihood ratio tests and *p*-values assessed model fit and nonlinearity. (3) Use RCS analysis to decide how to include independent variables in the binary logistic regression model: as categorical if nonlinear, or continuous if linear. (4) Multivariate-adjusted regression models: Model 1 examined crude associations between PM_2.5_ and its constituents with NSSI; Model 2 adjusted for sociodemographic and behavioral factors; Model 3 added environmental factors. Categorical variables were included in the model as dummy variables; continuous variables were included directly in the regression equation as continuous variables. (5) Mixed exposure effects were analyzed using weighted quantile sum (WQS) regression and Quantile G-Computation (Qgcomp). Previous toxicological evidence indicates that all five PM_2.5_ constituents exhibit adverse neurotoxic effects, and we observed a consistent positive association in the single-pollutant model. Therefore, we incorporated positive constraints into the WQS analysis. At the same time, we acknowledge that this assumption may oversimplify the actual dynamics of the mixture; consequently, we also employed Qgcomp as a complementary analytical method, which does not require directional constraints. (6) Stratification and Interaction Analysis. We examined interactions among potential effect-modifying factors were examined, including adolescents’ grade, gender, family’s economic situation, laerning burden, left behind, and biological rhythms. To explore the distinct regulatory effects of biological rhythms, we broke down the composite score into sleep, activity, eating, and digital media usage. Subgroup analyses were conducted for each dimension. With the exception of biological rhythms, all other subgroup analyses were exploratory analyses designed to identify potential heterogeneity of effects. No correction for multiple comparisons was performed; therefore, the results should be interpreted with caution, and undue reliance should not be placed on individual significant findings. (7) To verify the robustness of the results, we conducted several sensitivity analyses: First, we uniformly encoded all pollutants as continuous variables in increments of one interquartile range (IQR) to test the consistency of effect sizes under different coding schemes. Second, we replaced the annual average exposure window with the seasonal average for the three months prior to the survey to assess short-term exposure effects. Furthermore, we used the *E*-value analysis method to quantitatively assess the potential impact of unmeasured confounders on the results. Finally, we included temperature in the fully adjusted model and performed a regression analysis.

NSSI, a categorical variable with less than 2% missing data, was imputed using the mode method, assigning “None” to missing cases. All statistical analyses were performed using SPSS 23.0, R-software (version 4.4.1). The rms package is used for RCS, the gWQS package for WQS, and the qgcomp package for Qgcomp.

## 3 Results

### 3.1 Descriptive results

This study included 18,398 participants with a mean age of 15.49 ± 1.83 (SD) years, of whom 9,385 (51.01%) were male. Participants were divided into a NSSI group (*n* = 4,610) and a non-NSSI group (*n* = 13,788) based on the presence or absence of non-suicidal self-injury behavior. Baseline feature comparisons revealed statistically significant differences between groups in grade, gender, residence, father’s education level, monther’s education level, family’s economic situation, close pantners, learning burden, left behind, and biological rhythms (*p* < 0.05) (Table 1).

**Table 1:** Baseline characteristics of participants included in the study (*n* = 18,398).

Characteristic	Total	NSSI		*p*-value
	(*n* = 18,398)	Yes (*n* = 4,610)	No (*n* = 13,788)	
Grade, *n* (%)				0.01
Junior high school	8,669 (47.12)	2077 (45.05)	6,592 (47.81)	
High school	9,729 (52.88)	2,533 (54.95)	7,196 (52.19)	
Gender, *n* (%)				<0.01
Boy	9,385(51.01)	2091(45.36)	7,294(52.90)	
Girl	9,013(48.99)	2,519(54.64)	6,494(47.10)	
Residence, *n* (%)				<0.01
Rural	4,140(22.50)	1,133(24.58)	3,007(21.81)	
Town	14,258(77.50)	3,477(75.42)	10,781(78.19)	
Only child, *n* (%)				0.71
Yes	3,160(17.18)	800(17.35)	2,360(17.12)	
No	15,238(82.82)	3,810(82.65)	11,428(82.88)	
Father’s educational level, *n* (%)				<0.01
Primary school or below	3,473(18.88)	1,019(22.10)	2,454(17.80)	
Junior high school	5,807(31.56)	1,518(32.93)	4,289(31.10)	
High school or technical secondary school	4,507(24.50)	1,027(22.28)	3,480(25.24)	
College degree or above	4,611(25.06)	1,046(22.69)	3,565(25.86)	
Mother’s educational level, *n* (%)				<0.01
Primary school or below	4,382(23.82)	1,232(26.72)	3,150(22.84)	
Junior high school	5,526(30.04)	1,417(30.74)	4,109(29.80)	
High school or technical secondary school	4,173(22.68)	950(20.61)	3,223(23.38)	
College degree or above	4,317(23.46)	1,011(21.93)	3,306(23.98)	
Family’s economic situation, *n* (%)				<0.01
Poor	3,118(16.95)	1,002(21.74)	2,116(15.35)	
Medium	12,384(67.31)	2,921(63.36)	9,463(68.63)	
Good	2,896(15.74)	687(14.90)	2,209(16.02)	
Close partners, *n* (%)				<0.01
0	638(3.47)	267(5.79)	371(2.69)	
1–2	5,396(29.33)	1,560(33.84)	3,836(27.82)	
3–5	7,893(42.90)	1846(40.04)	6,047(43.86)	
≥6	4,471(24.30)	937(20.33)	3,534(25.63)	
Learning burden, *n* (%)				<0.01
Medium and below	10,624(57.75)	2,252(21.20)	8,372(78.80)	
Heavy	7,774(42.25)	2,358(30.33)	5,416(69.67)	
Life behind, *n* (%)				<0.01
Yes	4,379(23.80)	1,379(29.91)	3,000(21.76)	
No	14,019(76.20)	3,231(70.09)	10,788(78.24)	
Biological rhythms, *n* (%)				<0.01
Normal	4,379(23.80)	1,379(29.91)	3,000(21.76)	
Disorder	14,019(76.20)	3,231(70.09)	10,788(78.24)	

Table 2 displays the yearly average levels of PM_2.5_ and its constituents. The median concentration of PM_2.5_ was 27.78 μg/m^3^ (IQR: 23.2 μg/m^3^), while the median concentrations of the five constituents were: 1.41 μg/m^3^ (BC), 3.49 μg/m^3^ (NH₄^+^), 4.85 μg/m^3^ (NO₃^−^), 7.35 μg/m^3^ (OM), and 5.14 μg/m^3^ (SO₄^2−^). PM_2.5_ and its constituents showed significant positive correlations, with *Spearman* correlation coefficients (rs) ranging from 0.85 to 0.99 (*p* < 0.01) (Supplementary Table S1).

**Table 2:** Annual average concentrations of PM_2.5_ and its constituents.

Variables	Mean	SD	Median	Minimum	Maximum	IQR
PM_2.5_	31.49	11.69	27.78	7.24	53.99	23.2
BC	1.46	0.32	1.41	0.42	2.12	0.46
NH_4_^+^	3.64	1.82	3.49	0.74	7.45	2.75
NO_3_^−^	5.33	2.99	4.85	0.89	11.67	3.88
OM	7.44	1.69	7.35	1.98	11.22	2.03
SO_4_^2−^	5.44	1.46	5.14	1.39	8.89	1.89

### 3.2 Exposure-response curve for PM₂.₅ and its constituents

**Figure 1:**
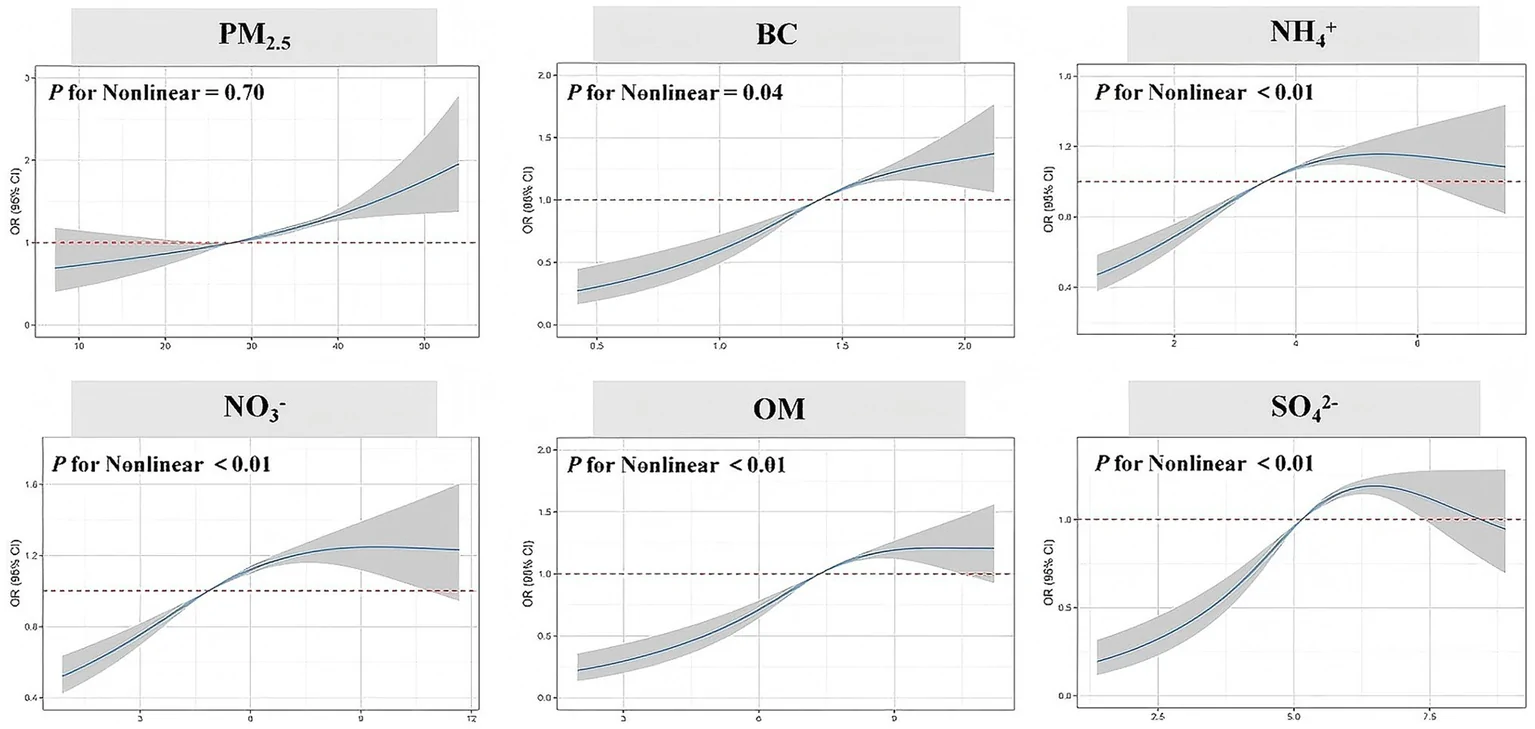
Restricted cubic spline for the associations between PM_2.5_ and its constituents and NSSI. Subplots represent PM_2.5_, BC, NH_4_^+^, NO_3_^−^, OM, SO_4_^2−^.

Figure 1 illustrates the dose–response link between PM₂.₅ and its constituents with NSSI after accounting for covariates These dose–response curves exhibit a consistent upward trend, indicating a positive correlation between PM₂.₅ and its constituents with non-suicidal self-injury. At low exposure levels, all pollutant curves show an approximately linear increase. As concentrations continue to rise, the association between non-suicidal self-injury and all constituents except PM₂.₅ gradually flattens or weakens. Nonlinearity tests supported these observations: *P*_nonlinear values for all constituents except PM₂.₅ were less than 0.01, indicating significant evidence of nonlinearity. Therefore, we included PM_2.5_ as a continuous variable in the model, while analyzing the remaining constituents as categorical variables.

### 3.3 Association between individual exposure to PM_2.5_ and its constituents and non-suicidal self-injury among chinese adolescents

Using three models of binary logistic regression, we comprehensively analyzed the association between individual exposures to PM_2.5_ and its constituents and non-suicidal self-injury among adolescents, as shown in Table 3. The association between each pollutant and non-suicidal self-injury remained statistically significant (*p* < 0.01) across models, from the crude model (Model 1) to progressively adjusted models (Model 2 and Model 3).

**Table 3:** Association between PM_2.5_ and its constituents and NSSI among Chinese adolescents.

Variables	Model 1		Model 2		Model 3	
	*β*	*OR* (95% *CI*)	*β*	*OR* (95% *CI*)	*β*	*OR* (95% *CI*)
PM_2.5_						
Continuous variable						
Per unit increase	0.02	1.02(1.01,1.02)^**^	0.02	1.02(1.02,1.02)^**^	0.02	1.02(1.02,1.03)^**^
BC						
Categorical variable						
Low exposure	Reference (0)		Reference (0)		Reference (0)	
High exposure	0.39	1.48(1.37,1.59)^**^	0.42	1.52(1.41,1.65)^**^	0.57	1.76(1.60,1.94)^**^
NH_4_^+^						
Categorical variable						
Low exposure	Reference (0)		Reference (0)		Reference (0)	
High exposure	0.38	1.46(1.35,1.57)^**^	0.31	1.36(1.26,1.48)^**^	0.36	1.44(1.30,1.58)^**^
NO_3_^−^						
Categorical variable						
Low exposure	Reference (0)		Reference (0)		Reference (0)	
High exposure	0.34	1.41(1.31,1.52)^**^	0.31	1.36(1.25,1.47)^**^	0.38	1.46(1.32,1.62)^**^
OM						
Categorical variable						
Low exposure	Reference (0)		Reference (0)		Reference (0)	
High exposure	0.36	1.43(1.33,1.55)^**^	0.37	1.45(1.34,1.57)^**^	0.49	1.63(1.47,1.79)^**^
SO_4_^2−^						
Categorical variable						
Low exposure	Reference (0)		Reference (0)		Reference (0)	
High exposure	0.39	1.47(1.37,1.59)^**^	0.32	1.38(1.27,1.50)^**^	0.38	1.46(1.32,1.61)^**^

^**^p < 0.01.

Model 1 did not undergo any adjustments.

Model 2 was adjusted for grade, gender, residence, father’s educational level, mother’s educational level, family’s economic situation, close partners, learning burden, life behind, and biological rhythms.

Model 3 was adjusted for grade, gender, residence, father’s educational level, mother’s educational level, family’s economic situation, close partners, learning burden, life behind, and biological rhythms, nighttime light exposure, NDVI, and O_3_ exposure.

In continuous variable analysis, a rise in PM_2.5_ mass concentration by each unit was notably linked to a higher risk of NSSI, yielding an *OR* of 1.02 (95% *CI*: 1.02–1.03) in the fully adjusted model (Model 3). Among the constituents, BC exhibited the strongest association: in categorical variable analysis, compared with the low BC exposure group, the risk of NSSI in the high BC exposure group increased to 1.76 times in Model 3 (95% *CI*: 1.60–1.94). When analyzed as dichotomous variables, NH₄^+^, NO₃^−^, OM, and SO₄^2−^ also demonstrated significant and robust positive associations with NSSI risk after stepwise adjustment for sociodemographic factors, biological rhythms, and environmental variables [NH₄^+^: *OR* = 1.44 (95% *CI*: 1.30–1.58), NO₃^−^: *OR* = 1.46 (95% *CI*: 1.32–1.62), OM: *OR* = 1.63 (95% *CI*: 1.47–1.79), SO₄^2−^: *OR* = 1.46 (95% *CI*: 1.32–1.61)].

### 3.4 Weighted analysis of PM_2.5_ constituents in mixed exposure among adolescents with non-suicidal self-injury

**Figure 2:**
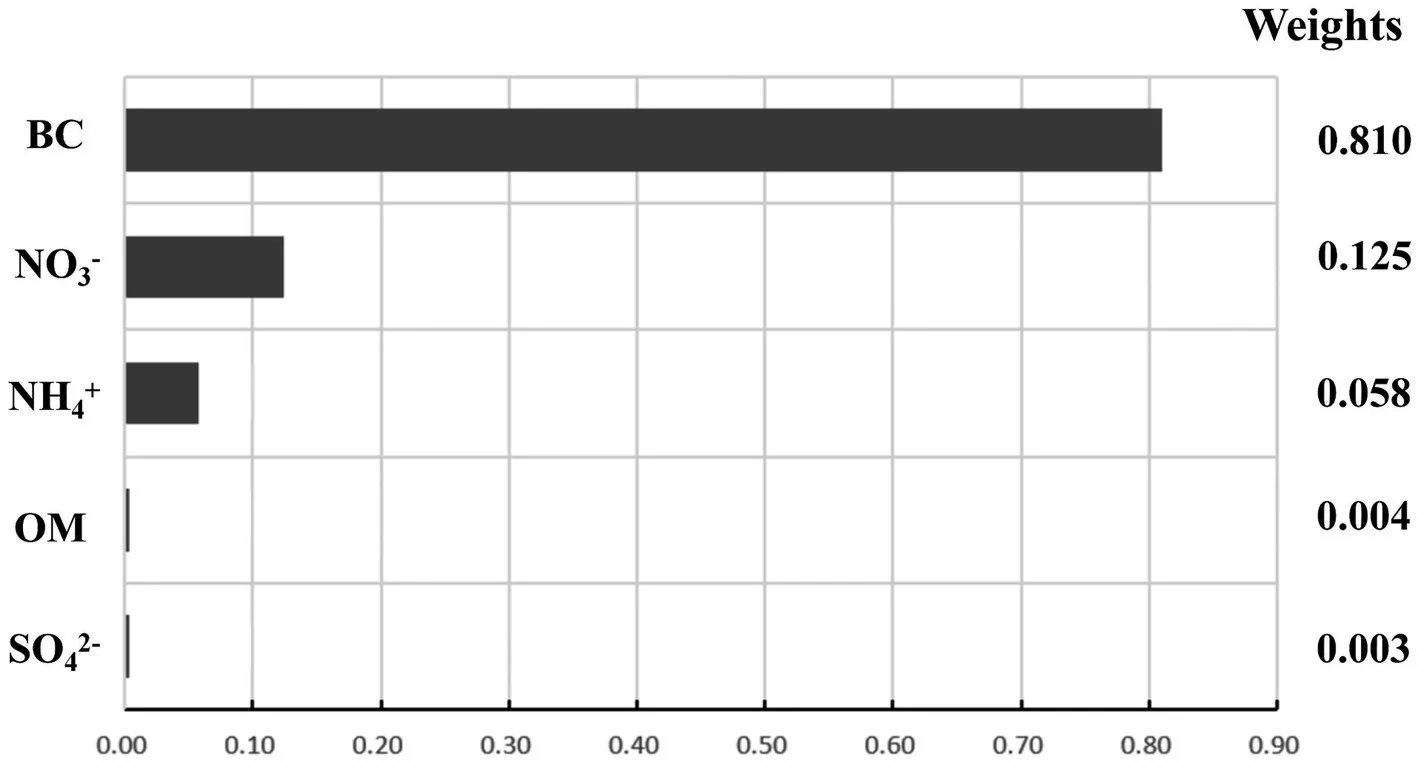
The association between PM_2.5_ constituents and NSSI based on WQS regression analysis.

In the WQS analysis, the contribution weights of PM_2.5_ constituents in the WQS index are shown in Figure 2, where BC is notably influential in the NSSI association with a weight of 0.810. The respective weights for NO₃^−^, NH₄^+^, OM, and SO₄^2−^ were 0.125, 0.058, 0.004, and 0.003. The Qgcomp analysis showed similar findings, with BC being the main component with the greatest weight in the NSSI association, showing an effect size of 0.671 (Supplementary Figure S2). The effect sizes for the remaining constituents NH₄^+^, NO₃^−^, SO₄^2−^, and OM were 0.203, 0.126, 0.549, and 0.451, respectively.

### 3.5 Stratified analysis

**Figure 3:**
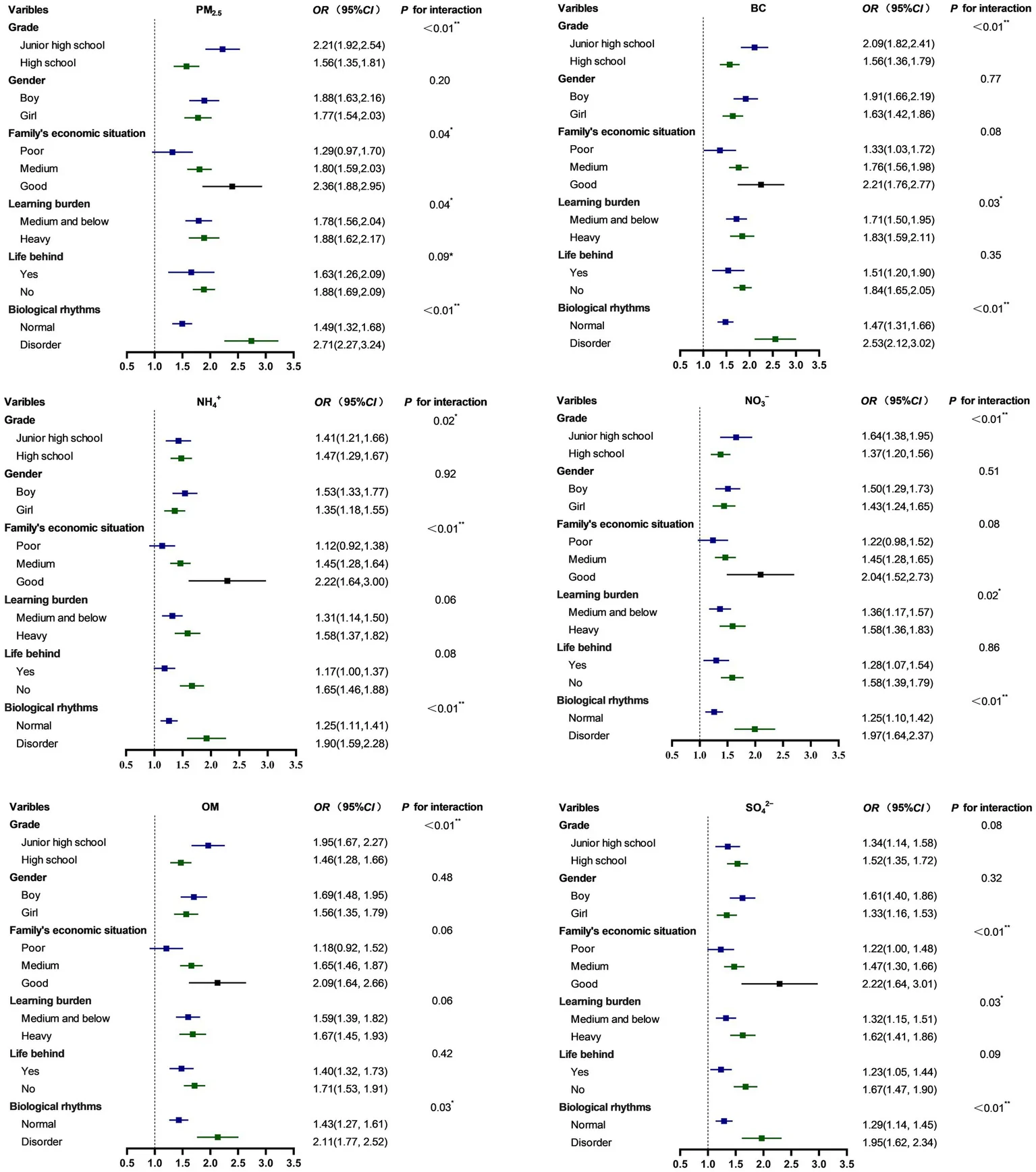
The associations between PM_2.5_ and its constituents concentrations and the likelihood of NSSI among multiple subgroups. The subgroups include grade, gender, family’s economic situation, learning burden, life behind, biological rhythms. The boxes represent ORs, with horizontal lines representing 95% *CI*. The black dashed line represents the reference line (*OR* = 1).

Stratified analysis based on grade, gender, family’s economic situation, learning burden, left behind, and biological rhythms examined the relationship between PM_2.5_ and its constituents with non-suicidal self-injury. Detailed results in Figure 3 indicate significant population-specific differences in pollutant effects on non-suicidal self-injury risk. Among these, biological rhythms exhibited the strongest risk-amplifying effect. The interaction term between biological rhythm disruption and PM₂.₅ was statistically significant (*P* for interaction = 0.01). In the stratified analysis, among adolescents with biological rhythm disruption, the risk of NSSI associated with PM₂.₅ exposure was significantly higher than among those with normal biological rhythms (*OR* = 2.71, 95% *CI*: 2.27–3.24). BC and other PM₂.₅ constituents also exhibited consistent and significant interaction patterns, further supporting the universal moderating effect of biological rhythms. Further analysis across various dimensions of biological rhythms (Supplementary Tables S2, S3) indicates that activity rhythm and eating rhythm exert a significant moderating effect on PM₂.₅ and black carbon pollution. This indicates that disruptions in these two rhythms substantially amplify the risk impact of pollutants on non-suicidal self-injury.

Furthermore, adolescents from good family economical situation exhibited higher risks across multiple pollutants (e.g., *OR* = 2.36 for PM_2.5_). Junior high students demonstrated greater sensitivity to pollutants like PM_2.5_, NO₃^−^, and BC compared to senior high students. Self-reported heavy learning burden also generally amplified the negative effects of pollutants. In contrast, interactions between gender and left-behind experience were not significant for any pollutant.

### 3.6 Sensitivity analyses

We standardized all pollutants as continuous variables measured per IQR increment and reran the regression analysis. The results (Supplementary Table S4) showed that all pollutants remained significantly positively correlated with the risk of NSSI. Under the uniform quartile-range scaling, the effect size for total PM_2.5_ is slightly larger than that for BC. The results of the short-term exposure analysis (Supplementary Figure S3; Supplementary Table S5) are generally consistent with our main findings. The *E*-value analysis (Supplementary Table S6) indicates that an unmeasured confounder would need to be associated with both PM_2.5_ constituent exposure and NSSI with a odds ratio of at least 2.92 (for BC) to fully explain the observed point estimate, suggesting that the observed associations are relatively robust to unmeasured confounding. After controlling for temperature as a confounding factor (Supplementary Table S7), the resulting odds ratios were consistent with the unadjusted results in terms of both direction and statistical significance.

## 4 Discussion

The study found that exposure to PM₂.₅ and its constituents was significantly and positively associated with elevated adolescent NSSI risk. Among the pollutant constituents included in the analysis, BC exhibited a stronger independent positive association and contributed the highest weight in the mixed exposure model. Stratified analyses further indicated that the adverse effects of PM₂.₅ and its constituents were more pronounced among adolescents with disrupted biological rhythms, from good family economic situations, attending junior high school, and reporting heavy learning burdens. To our knowledge, this is the first study to specify air pollution constituents, especially BC, as risk factors for NSSI in Chinese adolescents, highlighting the importance of biological rhythms. It offers evidence for creating an integrated intervention framework connecting environment, behavior, and mental health.

Our research aligns with studies that associate fine particulate pollution with mental health disorders (33, 34). Based on evidence from experimental and animal studies, several biologically plausible pathways may mediate this association. First, it induces oxidative stress and neuroinflammation through both direct and indirect pathways. Directly, fine particles like BC can penetrate the brain via the olfactory pathway. Indirectly, particles in the alveoli can enter the bloodstream, with inflammatory mediators crossing the blood–brain barrier (35). These processes generate significant reactive oxygen species (ROS) in the central nervous system, potentially causing oxidative stress and neuronal damage in brain areas like the prefrontal cortex and amygdala (36). This damage is hypothesized to be linked to impaired impulse control and challenges in managing negative emotions, which are fundamental to NSSI (37, 38). Moreover, oxidative stress activates microglia, triggering inflammatory pathways like NF-κB, which exacerbate neuroinflammation and disrupt emotional regulation (39). This could makes individuals more likely to use NSSI to quickly cope with negative emotions (40, 41).

Second, interference with the neuroendocrine system may disrupts hormonal balance and neurochemical stability. Fine particulate matter exposure has been shown in population studies to activates the hypothalamic–pituitary–adrenal (HPA) axis, altering hormone secretion rhythms like cortisol, which can impair cognitive and emotional regulation (42). Moreover, this heightened HPA activity is linked to emotional instability and increased suicide risk (43). Third, PM_2.5_ and its constituents change the volume and composition of adolescent amygdala subregions, weakening its executive control and reward system (44, 45). This could contribute to diminished impulse control in adolescents while simultaneously reducing the pain stimulus associated with self-harm, thereby increasing the risk of individuals resorting to extreme coping mechanisms such as NSSI (46).

Finally, BC is one of the key cconstituents of PM_2.5_, and China has consistently been a major contributor to global anthropogenic black carbon emissions (47). Compared to other constituents, the ultrafine particle size and elevated surface activity of BC enhance its ability to traverse physiological barriers and access the central nervous system. BC can enter the brain directly via the olfactory nerve, the blood–brain barrier, or macrophage-mediated transport, subsequently inducing the overproduction of ROS. The resulting oxidative stress can disrupt serotonin metabolism and disturb neurochemical homeostasis, both of which are closely implicated in emotional regulation and impulse control. It should be noted, however, that much of this mechanistic evidence derives from experimental models; direct evidence in adolescent populations remains limited. Concurrently, BC can activate the NLRP3/NLRC4 inflammasome, triggering the caspase-1/IL-1β cascade and significantly amplifying neuroinflammation (48, 49). Collectively, these pathways may contribute to neurobiological alterations that increase vulnerability to self-injurious behaviors. Furthermore, the polycyclic aromatic hydrocarbons and heavy metals adsorbed onto these particles exhibit neurotoxic characteristics, which may synergistically disrupt the dopaminergic system and affect impulsive behavior (50, 51). Particularly noteworthy is research on student populations indicating that BC exhibits the highest deposition fraction and deposition dose in the head region, suggesting this group may face more direct and significant risks of neurological exposure (52).

In this study, the quantitative differences in component weights between WQS and Qgcomp stem primarily from differences in their underlying algorithmic principles, rather than from conflicting results. By constructing a single weighted index among highly collinear exposure variables, WQS tends to attribute common variance to the dominant component (BC), thereby reducing the independent weights of other components strongly correlated with it; In contrast, Qgcomp preserves the marginal contributions of each component, thereby reflecting synergistic effects in the mixture more evenly. Given that the positive constraint in WQS may oversimplify the complexity of real-world exposures, we interpret the component weights with caution. Due to high collinearity among components and BC’s established role as a combustion tracer, current statistical methods cannot separate BC’s independent effect from the entire combustion-related mixture. Thus, we view BC mainly as a strong indicator of the harmful effects of combustion pollution, rather than an independent causal factor.

This study further reveals an interactive relationship between exposure to PM_2.5_ and its constituents and biological rhythm disruption. On one hand, PM_2.5_ acts on the suprachiasmatic nucleus of the hypothalamus, disrupting melatonin secretion and cortisol rhythms to directly induce emotional dysregulation while further exacerbating biological rhythm disturbances (42, 53). On the other hand, pre-existing biological rhythm disruption reduces the body’s antioxidant and anti-inflammatory capabilities, heightening neural tissue sensitivity to PM_2.5_ toxicity and amplifying its damaging effects on neuroinflammation and oxidative stress (54).

Additionally, disruptions in activity rhythm and eating rhythm can heighten the impact of PM_2.5_ and BC on adolescent NSSI. On one hand, school schedules characterized by weekdays significantly increase adolescents outdoor activity during peak air pollution periods (e.g., 07:00–09:00 and 16:00–18:00) (55). Moreover, as adolescents age, their participation in extracurricular tutoring and social activities significantly increases (56). Their exposure to pollution rises, leading to stress and reduced behavioral regulation, which heightens vulnerability to NSSI. On the other hand, irregular eating patterns may interact with PM_2.5_ exposure through the microbiome-gut-brain axis. Animal studies show that PM_2.5_ disrupts gut microbiota, while irregular eating affects gut rhythms and neuroendocrine balance (57). Together, these factors worsen neuroinflammation and neurotransmitter issues, reducing emotional stability and behavioral control. Therefore, activity and eating rhythms not only can indicate healthy behaviors, but also influence the relationship between environmental pollution and mental health.

Although there was no notable interaction found between sleep rhythm and the impact of PM_2.5_ or BC on NSSI, this does not necessarily negate their importance. It more likely reflects limitations in the current study’s measurement precision and exposure scenario specificity. First, questionnaire assessments may struggle to capture subtle effects of air pollution on sleep physiological indicators such as sleep latency, efficiency, and nighttime awakenings—metrics proven to be more sensitive to pollution-induced neuroinflammation (58). Second, from a behavioral functional perspective, sleep rhythm disorders are often highly comorbid with excessive use of electronic devices, reduced daytime activity, and irregular eating patterns, and there are functional interrelationships among these dimensions at the behavioral level. Additionally, adolescents spend most of their sleep time indoors, where actual exposure levels result from combined outdoor infiltration and indoor sources. This error in the exposure estimate could potentially contribute to the true effect size of the sleep-modulating effect. The absence of real-time exposure data at the individual or bedroom level may obscure variations in nocturnal exposure (59, 60). Future research should therefore integrate more precise sleep monitoring (e.g., actigraphy) with personalized real-time exposure assessment to clarify the role of sleep rhythm in linking environmental factors to mental health.

The study reveals that adolescents from families with good economic situations are at a higher risk of NSSI, challenging common beliefs. This risk may be due to their proximity to urban pollution and the psychological stress of high expectations, showing that economic advantage does not protect against mental health issues. The developmental stage represents a critical biological window. Junior high students generally exhibit higher sensitivity to PM₂.₅, BC and other pollutants than senior high students. This heightened vulnerability is closely linked to the incomplete myelination and functional immaturity of their higher-order regulatory regions, such as the prefrontal cortex (61, 62). Moreover, perceived heavy academic burden clearly demonstrates the “synergistic effect” between psychological and environmental stressors. Learning burden may synergize with the neurotoxicity of PM₂.₅, lowering the threshold for self-behavioral regulation. Adolescents with a high learning burden are particularly at risk of NSSI. This underscores the need for targeted public health and educational interventions.

Four essential aspects summarize the primary strengths of this study. First, it is the first to systematically explore the link between PM_2.5_ constituents and adolescent NSSI in a large sample, filling a research gap; Second, it analyzes the specific impact of each constituent for a deeper understanding of pollutants’ effects on adolescent mental health; Third, it uses a time-weighted exposure model considering both home and school locations for accurate pollution exposure assessment; Fourth, it identifies vulnerable groups through demographic and biological rhythm-based stratified analysis, highlighting differences in exposure among adolescents.

This study also has several limitations. First, this study utilized a cross-sectional design to examine NSSI incidents and PM_2.5_ exposure only in the 12 months preceding the survey, without noting the dates of initial episodes. Consequently, it cannot determine the timeline between PM_2.5_ and its constituents exposure and NSSI, making it unable to establish causality or dismiss reverse causality. Furthermore, while the SBRDA questionnaire covers the past 30 days and can capture recent stability patterns, this difference in time scale still limits the precise identification of the transient dynamics of exposure-modification effects. Second, our exposure assessment was based solely on participants’ home and school addresses and did not account for other activity settings (e.g., commuting routes, leisure venues), which may introduce measurement errors in exposure estimation. Additionally, the 10-kilometer resolution of the environmental data platform might miss detailed spatial variations of pollutants like black carbon in urban areas. Despite this, the grid-based segmentation does not bias the NSSI results and generally biases effect estimates towards zero. Thus, our observed associations are probably conservative, not exaggerated. Third, NSSI data are self-reported, which may lead to recall or social desirability bias. Furthermore, this study employed a simple binary classification analysis for the outcomes; future research should utilize large-sample prospective cohorts to further explore the differential responses of different NSSI subtypes and frequency gradients to environmental pollutants. Fourth, due to practical constraints in data collection, we were unable to obtain information on several potentially important confounders, such as parents’ mental health status and detailed indoor air quality parameters. Fifth, the study focused on key PM_2.5_ chemical constituents, omitting other harmful components like heavy metals and polycyclic aromatic hydrocarbons, as well as interactions with other air pollutants. Comprehensive future assessments are needed. Sixth, given the differences across regions in the chemical constituents of PM_2.5_ (e.g., coal-fired heating in the north versus a greater contribution from motor vehicles in the south), the incident of NSSI, and adolescents’ daily activity patterns, the results of this study should be interpreted in light of local conditions when extrapolating them.

## 5 Conclusion

This study demonstrates that exposure to PM₂.₅, particularly BC, is associated with a higher likelihood of NSSI among Chinese adolescents. Disrupted biological rhythm may act as a key amplifying factor in this pathway. These findings underscore the vulnerability of middle school students, who may be particularly sensitive to the mental health effects of ambient air pollution. Specifically, feasible interventions include scheduling outdoor activities according to real-time air quality index, installing air purifiers in classrooms in heavily polluted areas, and integrating rhythm-maintenance behaviors (regular meals, physical activity, and limited pre-bedtime screen use) into health education, reinforced through home-school collaboration. Although the current study’s conclusions offer preliminary epidemiological insights and hypotheses about a possible association. Future prospective cohort studies, along with mechanism experiments, are needed to clarify the temporal relationship between exposure and self-harm behavior and to further verify the potential causal connection.

## Data Availability

The original data used and analyzed in the study are available via the corresponding author on reasonable request.

## Footnotes

1
http://tapdata.org.cn

2
https://www.earthdata.nasa.gov/

3
https://eogdata.mines.edu/products/vnl/
