# Supplementary material for: Association between PM2.5 constituents and non-suicidal self-injury among Chinese adolescents: modifying role of biological rhythm
Source: Front Public Health. 2026 Aug 20;14:1887542. doi: 10.3389/fpubh.2026.1887542 (PMC13538845; doi:10.3389/fpubh.2026.1887542)
Supplement: Supplementary file 1 [file Data_Sheet_1.docx]

***Supplement***

**Fig. S1.** Flow diagram for the inclusion of the study population.

**Fig. S2.** The association between the main constituents of PM_2.5_ and NSSI based on Qgcomp analysis.

**Fig S3. Restricted cubic spline for the associations between short-term exposure to PM_2.5_ and its constituents and NSSI.** Subplots represent PM_2.5_ , BC , NH_4_^+^ , NO_3_^-^ , OM , SO_4_^2-^.

**Table S1 Correlation analysis of annual average concentrations of PM_2.5_ and its constituents.**

|  | PM_2.5_ | BC | NH_4_^+^ | NO_3_^−^ | OM | SO_4_^2−^ |
| --- | --- | --- | --- | --- | --- | --- |
| PM_2.5_ | 1 | 0.94^**^ | 0.94^**^ | 0.95^**^ | 0.95^**^ | 0.88^**^ |
| BC | 0.94^**^ | 1 | 0.85^**^ | 0.88^**^ | 0.99^**^ | 0.89^**^ |
| NH_4_^+^ | 0.94^**^ | 0.85^**^ | 1 | 0.97^**^ | 0.88^**^ | 0.94^**^ |
| NO_3_^−^ | 0.95^**^ | 0.88^**^ | 0.97^**^ | 1 | 0.91^**^ | 0.89^**^ |
| OM | 0.95^**^ | 0.99^**^ | 0.88^**^ | 0.91^**^ | 1 | 0.90^**^ |
| SO_4_^2−^ | 0.88^**^ | 0.89^**^ | 0.94^**^ | 0.89^**^ | 0.90^**^ | 1 |

^**^: *P* ＜ 0.01

**Table S2**  Stratified analysis of the association between PM_2.5_ and NSSI across different biological rhythms dimensions.

| **Categorical variables** | ***OR* (95% *CI*)** | ***P* for interaction** |
| --- | --- | --- |
| **Sleep rhythm** |  | 0.16 |
| Normal | 1.66(1.47,1.87) |  |
| Disorder | 2.15(1.81,2.55) |  |
| **Activity rhythm** |  | 0.02^*^ |
| Normal | 1.68(1.50,1.89) |  |
| Disorder | 2.63(2.17,3.19) |  |
| **Eating rhythm** |  | ＜0.01^**^ |
| Normal | 1.57(1.40,1.77) |  |
| Disorder | 2.59(2.15,3.11) |  |
| **Digital media usage** |  | 0.73 |
| Normal | 1.74(1.55,1.95) |  |
| Disorder | 2.08(1.72,2.51) |  |

**Table S3**  Stratified analysis of the association between BC and NSSI across different biological rhythms dimensions.

| **Categorical variables** | ***OR* (95% *CI*)** | ***P* for interaction** |
| --- | --- | --- |
| **Sleep rhythm** |  | 0.16 |
| Normal | 1.60(1.43,1.80) |  |
| Disorder | 2.09(1.77,2.48) |  |
| **Activity rhythm** |  | 0.02^*^ |
| Normal | 1.61(1.44,1.80) |  |
| Disorder | 2.57(2.13,3.11) |  |
| **Eating rhythm** |  | ＜0.01^**^ |
| Normal | 1.55(1.38,1.74) |  |
| Disorder | 2.34(1.95,2.80) |  |
| **Digital media usage** |  | 0.73 |
| Normal | 1.69(1.51,1.89) |  |
| Disorder | 2.01(1.67,2.43) |  |

**Table S4 Association between long-term exposure to PM_2.5_ and its constituents and NSSI among middle chinese adolescents.**

| Varibles | Model 1 | | Model 2 | | Model 3 | |
| --- | --- | --- | --- | --- | --- | --- |
|  | *β* | *OR* (95% *CI*) | *β* | *OR* (95% *CI*) | *β* | *OR* (95% *CI*) |
| PM_2.5_ |  | | | | | |
| Per IQR increase | 0.41 | 1.50(1.40,1.60)^**^ | 0.44 | 1.55(1.44,1.66)^**^ | 0.54 | 1.71(1.57,1.86)^**^ |
| BC |  | | | | | |
| Per IQR increase | 0.29 | 1.33(1.27,1.40)^**^ | 0.30 | 1.35(1.28,1.42)^**^ | 0.37 | 1.45(1.36,1.54)^**^ |
| NH_4_^+^ |  | | | | | |
| Per IQR increase | 0.34 | 1.40(1.33,1.47)^**^ | 0.33 | 1.39(1.32,1.47)^**^ | 0.39 | 1.47(1.38,1.57)^**^ |
| NO_3_^−^ |  | | | | | |
| Per IQR increase | 0.28 | 1.32(1.26,1.37)^**^ | 0.27 | 1.32(1.25,1.38)^**^ | 0.34 | 1.41(1.33,1.49)^**^ |
| OM |  | | | | | |
| Per IQR increase | 0.24 | 1.27(1.22,1.32)^**^ | 0.24 | 1.27(1.22,1.33)^**^ | 0.30 | 1.35(1.28,1.42)^**^ |
| SO_4_^2−^ |  | | | | | |
| Per IQR increase | 0.26 | 1.30(1.24,1.35)^**^ | 0.25 | 1.29(1.23,1.35)^**^ | 0.29 | 1.34(1.26,1.42)^**^ |

Abbreviations: **:*P* ＜ 0.01.

Model 1 did not undergo any adjustments.

Model 2 was adjusted for grade, gender, residence, father’s educational level, mother’s educational level, family’s economic situation, close partners, learning burden, life behind, and biological rhythms.

Model 3 was adjusted for grade, gender, residence, father’s educational level, mother’s educational level, family’s economic situation, close partners, learning burden, life behind, and biological rhythms, nighttime light exposure, NDVI, and O3 exposure.

**Table S5 Association between short-term exposure to PM_2.5_ and its constituents and NSSI among chinese adolescents.**

| Varibles | Model 1 | | Model 2 | | Model 3 | |
| --- | --- | --- | --- | --- | --- | --- |
|  | *β* | *OR* (95% *CI*) | *β* | *OR* (95% *CI*) | *β* | *OR* (95% *CI*) |
| PM_2.5_ |  | | | | | |
| Continuous variable |  | | | | | |
| Per unit increase | 0.02 | 1.02(1.02,1.02)^**^ | 0.02 | 1.02(1.02,1.03)^**^ | 0.02 | 1.03(1.02,1.03)^**^ |
| Per IQR increase | 0.39 | 1.48(1.39,1.59)^**^ | 0.44 | 1.55(1.44,1.67)^**^ | 0.47 | 1.61(1.48,1.74)^**^ |
| BC |  | | | | | |
| Continuous variable |  | | | | | |
| Per unit increase | 0.73 | 2.08(1.80,2.40)^**^ | 0.79 | 2.20(1.89,2.57)^**^ | 0.98 | 2.67(2.20,3.23)^**^ |
| Per IQR increase | 0.34 | 1.40(1.32,1.50)^**^ | 0.37 | 1.45(1.35,1.55)^**^ | 0.46 | 1.59(1.45,1.73)^**^ |
| NH_4_^+^ |  | | | | | |
| Continuous variable |  | | | | | |
| Per unit increase | 0.14 | 1.15(1.13,1.18)^**^ | 0.14 | 1.15(1.12,1.18)^**^ | 0.15 | 1.16(1.13,1.19)^**^ |
| Per IQR increase | 0.38 | 1.46(1.38,1.54)^**^ | 0.37 | 1.45(1.36,1.55)^**^ | 0.40 | 1.49(1.38,1.60)^**^ |
| Categorical variable |  | | | | | |
| Low exposure | Reference (0) | | Reference (0) | | Reference (0) | |
| High exposure | 0.38 | 1.47(1.36,1.58)^**^ | 0.32 | 1.37(1.26,1.49)^**^ | 0.39 | 1.48(1.35,1.63)^**^ |
| NO_3_^−^ |  | | | | | |
| Continuous variable |  | | | | | |
| Per unit increase | 0.08 | 1.08(1.07,1.10)^**^ | 0.08 | 1.08(1.07,1.10)^**^ | 0.09 | 1.09(1.08,1.11)^**^ |
| Per IQR increase | 0.28 | 1.32(1.27,1.38)^**^ | 0.28 | 1.32(1.26,1.39)^**^ | 0.32 | 1.37(1.30,1.45)^**^ |
| OM |  | | | | | |
| Continuous variable |  | | | | | |
| Per unit increase | 0.13 | 1.14(1.11,1.17)^**^ | 0.14 | 1.15(1.20,1.18)^**^ | 0.17 | 1.19(1.15,1.22)^**^ |
| Per IQR increase | 0.23 | 1.26(1.20,1.31)^**^ | 0.24 | 1.27(1.21,1.33)^**^ | 0.30 | 1.34(1.27,1.42)^**^ |
| SO_4_^2−^ |  | | | | | |
| Continuous variable |  | | | | | |
| Per unit increase | 0.18 | 1.20(1.16,1.24)^**^ | 0.17 | 1.19(1.15,1.24)^**^ | 0.19 | 1.20(1.15,1.26)^**^ |
| Per IQR increase | 0.26 | 1.30(1.24,1.36)^**^ | 0.25 | 1.28(1.22,1.36)^**^ | 0.27 | 1.31(1.23,1.40)^**^ |

Abbreviations: **:*P* ＜ 0.01.

Model 1 did not undergo any adjustments.

Model 2 was adjusted for grade, gender, residence, father’s educational level, mother’s educational level, family’s economic situation, close partners, learning burden, life behind, and biological rhythms.

Model 3 was adjusted for grade, gender, residence, father’s educational level, mother’s educational level, family’s economic situation, close partners, learning burden, life behind, and biological rhythms, nighttime light exposure, NDVI, and O3 exposure.

**Table S6 *E*-values for the association between long-term PM2.5 constituents exposure and NSSI.**

| Varibles | *E*-values (lower bound) |
| --- | --- |
| BC | 2.92 (2.58) |
| NH_4_^+^ | 2.24 (1.92) |
| NO_3_^-^ | 2.28 (1.97) |
| OM | 2.66 (2.30) |
| SO_4_^2-^ | 2.28 (1.97) |

**Table S6 Association between long-term exposure to PM_2.5_ and its constituents and NSSI among chinese adolescents.**

| Varibles | Model 3 | |
| --- | --- | --- |
|  | *β* | *OR* (95% *CI*) |
| PM_2.5_ |  | |
| Continuous variable |  |  |
| Per unit increase | 0.03 | 1.03(1.02,1.04)^**^ |
| BC |  | |
| Categorical variable |  | |
| Low exposure | Reference (0) | |
| High exposure | 0.35 | 1.42(1.25,1.60)^**^ |
| NH_4_^+^ |  | |
| Categorical variable |  | |
| Low exposure | Reference (0) | |
| High exposure | 0.20 | 1.22(1.10,1.35)^**^ |
| NO_3_^−^ |  | |
| Categorical variable |  | |
| Low exposure | Reference (0) | |
| High exposure | 0.14 | 1.15(1.03,1.30)^*^ |
| OM |  | |
| Categorical variable |  | |
| Low exposure | Reference (0) | |
| High exposure | 0.24 | 1.27(1.13,1.43)^**^ |
| SO_4_^2−^ |  | |
| Categorical variable |  | |
| Low exposure | Reference (0) | |
| High exposure | 0.22 | 1.24(1.12,1.38)^**^ |

Abbreviations: ^**^:*P* ＜ 0.01, ^*^:*P* ＜ 0.05.

Model 3 was adjusted for grade, gender, residence, father’s educational level, mother’s educational level, family’s economic situation, close partners, learning burden, life behind, and biological rhythms, nighttime light exposure, NDVI, O3 exposure, and temperature exposure.
